# Rhus Aromatica in Enuresis

**Published:** 1880-07-20

**Authors:** J. L. Lallerstedt

**Affiliations:** Georgia


### Southern Medical Record.

EDITORS:

T. S. POWELL, M.D. W. T. GOLDSMITH, M.D. R. C. WORD, M.D.

*R. C. WORD, M.D., Managing Editor.*

☞All Communications and Letters on Business connected with the RECORD must be addressed to the Managing Editor.

VOL. X. ATLANTA, GA., JULY 20, 1880. No. 7.

### ORIGINAL AND SELECTED ARTICLES.

### RHUS AROMATICA IN ENURESIS.

BY J. L. LALLERSTEDT, M.D., OF GEORGIA.

We who live in the country are frequently called upon to treat cases of enuresis in children, and, heretofore, the treatment has been very unsatisfactory both to the medical man and patient. But I am of the impression that we have now a remedy for this troublesome affection, and that is *rhus aromatica* (sweet sumac). I have only had three cases thus far to try it on.

During the month of December, 1878, a negro boy about 13 years of age came to see me and said as long as he would sit up he was not troubled about passing his urine, but as soon as he lay down he would "wet all over everything." I gave him rhus aromatica, twenty drops every three hours in a little water, last dose on going to bed. After taking it for one day he was able to lay down and sleep without the usual flow. After it gave out he was so much better, he thought he was well, but in a few days the same state of things returned. He came back and I gave him same quantity of the rhus with orders when it was out to come and get more, which he did. The third half ounce made a complete cure.

About that time I was consulted by a gentleman with regard to his son who had to get up from eight to ten times per night, and he was all bloated up and unable to do anything; could scarcely get about home for want of breath.

I gave him :

| | |
|---|---|
| Rhus aromatica | ʒ v. |
| Simple syrup | ℥ iij. |
| Water to make | ℥ v. |

Dose, one teaspoonful every three hours, and last dose on going to bed.

He only had to take some three or four bottles of above when he was able to do about as much as any one of his age on the farm. He is now in good health.

The third case was that of a lady who complained of passing from one to two gallons of urine during twenty-four hours, and most of it at night; yet she appeared in good health and was always able to attend to her household affairs. I put her upon half drachm doses of rhus aromatica three times a day, which gave her permanent relief after two days use of the remedy.

On case second I changed from simple syrup to glycerine.

I give you these notes for what they may be worth to the profession. I hope others will test the virtues of this medicine and report results.

